# PREVENTING FALLS AMONG OLDER FALLERS: LIVE-LIFE

**DOI:** 10.1093/geroni/igz038.3329

**Published:** 2019-11-08

**Authors:** Sarah L Szanton, Lindy Clemson, Minhui Liu, Laura N Gitlin, David L Roth, Melissa D Hladek, Sarah LaFave, Marianne Granbom

**Affiliations:** 1 Johns Hopkins University, Baltimore, Maryland, United States; 2 University of Sydney, Sydney, Australia, Australia; 3 Johns Hopkins University School of Nursing, Baltimore, Maryland, United States; 4 Drexel University, Philadephia, Pennsylvania, United States; 5 Lund University, Lund, Sweden, Sweden

## Abstract

OBJECTIVES: To evaluate whether a fall prevention intervention, adapted from the LiFE program, reduces fall risk in older adults who have previously fallen. DESIGN: Randomized controlled pilot trial SETTING: Participants’ homes INTERVENTION: LIVE-LIFE is an occupational therapy delivered fall prevention intervention that integrates strength and balance training into daily habits in 8 visits over 12 weeks. The intervention also provides 1) up to $500 in home safety changes prioritized by the participants 2) vision contrast screening and referral, and 3) personalized fall risk medication recommendations to Primary Care Providers (PCP) from a Pharmacist. This multi-component intervention was compared to a control condition consisting of CDC fall prevention materials and an individualized fall risk summary. MEASUREMENT: Primary outcome: Fall risk measured by Timed Up and Go (TUG) and Tandem stand. Secondary outcomes: Falls efficacy, feasibility and acceptability of the intervention. RESULTS: The sample of 37 people was 65% female, 65% white and an average 77 years old. Two were lost to follow up (95% retention). Compared to the control group, the mean of each outcome improved in the intervention. The LIVE-LiFE intervention had a large effect size (1.1) for amount of time study participants could hold a tandem stand, a moderate effect (0.5) in falls efficacy, and a small effect (0.1) in the TUG. CONCLUSION: LIVE-LIFE was acceptable to participants, feasible to provide, and averaged large to small effect sizes. Simultaneously addressing preventable fall risk factors is feasible and should be investigated due to the growing population at risk for falls.

